# Plasmalogenic Lipid Analogs as Platelet-Activating Factor Antagonists: A Potential Novel Class of Anti-inflammatory Compounds

**DOI:** 10.3389/fcell.2022.859421

**Published:** 2022-04-12

**Authors:** Pu Rong, Jie-Li Wang, Angelina Angelova, Zakaria A. Almsherqi, Yuru Deng

**Affiliations:** ^1^ Wenzhou Institute, University of Chinese Academy of Sciences, Wenzhou, China; ^2^ CNRS, Institut Galien Paris-Saclay, Université Paris-Saclay, Châtenay-Malabry, France; ^3^ Department of Physiology, Yong Loo Lin School of Medicine, National University of Singapore, Singapore, Singapore

**Keywords:** ether lipids, plasmalogen, platelet-activating factor, anti-PAF, anti-inflammation

## Abstract

Plasmalogens and Platelet-Activating Factor (PAF) are both bioactive ether phospholipids. Whereas plasmalogens are recognized for their important antioxidant function and modulatory role in cell membrane structure and dynamics, PAF is a potent pro-inflammatory lipid mediator known to have messenger functions in cell signaling and inflammatory response. The relationship between these two types of lipids has been rarely studied in terms of their metabolic interconversion and reciprocal modulation of the pro-inflammation/anti-inflammation balance. The vinyl-ether bonded plasmalogen lipid can be the lipid sources for the precursor of the biosynthesis of ether-bonded PAF. In this opinion paper, we suggest a potential role of plasmalogenic analogs of PAF as modulators and PAF antagonists (anti-PAF). We discuss that the metabolic interconversion of these two lipid kinds may be explored towards the development of efficient preventive and relief strategies against PAF-mediated pro-inflammation. We propose that plasmalogen analogs, acting as anti-PAF, may be considered as a new class of bioactive anti-inflammatory drugs. Despite of the scarcity of available experimental data, the competition between PAF and its natural plasmalogenic analogs for binding to the PAF receptor (PAF-R) can be proposed as a mechanistic model and potential therapeutic perspective against multiple inflammatory diseases (*e.g.,* cardiovascular and neurodegenerative disorders, diabetes, cancers, and various manifestations in coronavirus infections such as COVID-19).

## Introduction

**FIGURE 1:**
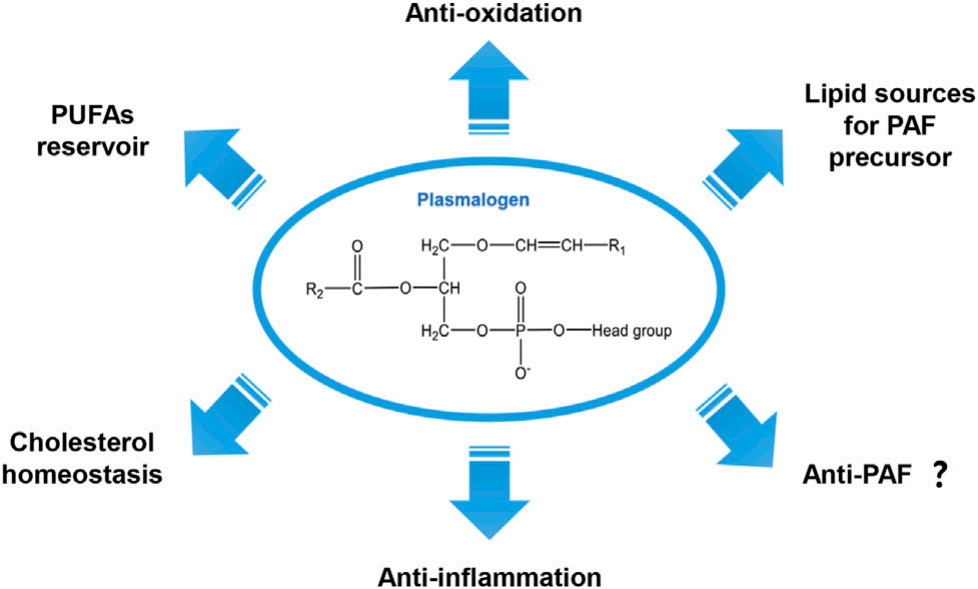
General chemical structure of the vinyl-ether bonded plasmalogen lipid and its proposed biological functions.

The interest in new classes of lipid-based anti-inflammatory drugs constantly increases in view of their critical role in the strategies to inhibit the inflammatory component of the coronavirus SARS-CoV-2 (severe acute respiratory syndrome-coronavirus-2) infection (Casari et al., 2021; Deng and Angelova, 2021; Schwarz et al., 2021), modulation of respiratory distress diseases (Mirastschijski et al., 2020; Zhuo et al., 2021) as well as in cancer and diabetes (Paul et al., 2019), and neuro-inflammation (Ifuku et al., 2012). Plasmalogens exert anti-inflammatory effects and have been first described by Feulgen and Voit in 1924 in relation to their characteristic production of aldehydes in acidic environment (Rapport, 1984). Plasmalogens are an unique type of ether glycerophospholipids carrying a vinyl-ether bond at sn-1 position of the glycerol backbone (Dorninger et al., 2020; Eiriksson et al., 2018) and typically a very long polyunsaturated fatty acid (PUFA) chain at sn-2 position (Figure 1). The head group usually comprises ethanolamine or choline at sn-3 position of glycerol backbone, which distinguishes plasmalogen phosphatidylethanolamine (pPE, or PE[P]) and plasmalogen phosphatidylcholine (pPC, or PC[P]) derivatives respectively (Dorninger et al., 2020). The levels of pPE often predominate over those of pPC (Paul et al., 2019). Of note, pPE is abundant in brain, especially in gray matter and white matter (Naughton and Trewhella, 1984; Lessig and Fuchs, 2009), whereas pPC is highly enriched in heart and skeletal muscles (Lessig and Fuchs, 2009; Braverman and Moser, 2012). Various biological functions have been proposed for plasmalogens including their protective role against oxidative damage as well as modulatory role in cell membrane structure and dynamics (Braverman and Moser, 2012; Almsherqi, 2021). In addition, plasmalogen deficiency has been reported to be associated with multiple diseases categorized as chronic inflammation triggered by oxidative stress (Pham et al., 2021).

Platelet activating factor (PAF), also known as acetyl-glyceryl-ether-phosphorylcholine, is an ether phosopholipid which is a potent lipid chemical mediator of inflammation (Demopoulos et al., 1979). PAFs are a family of endogenous pro-inflammatory lipids that may trigger many inflammatory and allergic responses (Palur Ramakrishnan et al., 2017). Noteworthy, plasmalogens can be the lipid sources for the precursor of PAF in lipid biosynthesis (Dorninger et al., 2020). However, the relationship between these two lipid species has been rarely studied in terms of their metabolic interconversion and reciprocal modulation in inflammation/anti-inflammation processes. The plasmalogenic analogs of PAF have been first introduced by Kulikov and Muzia (1992), referring to a family of molecules with similar chemical structures to plasmalogen or PAF. Compounds that inhibit PAF function are referred to as PAF antagonists (anti-PAF) and they may act as potential anti-inflammatory agents (Lordan et al., 2019). In this work, we argue and discuss the potential role of plasmalogenic analogs of PAF as PAF antagonists (anti-PAF). In our opinion, targeting the PAF receptor (PAF-R) by plasmalogenic analogs of PAF (anti-PAF) may provide an alternative strategy in the prevention and therapy for inflammation-mediated diseases.

## Significance of Plasmalogens as Bioactive Vinyl-Ether Lipids

Plasmalogens represent approximately one in five phospholipids in mammalian and human tissues, and they are particularly abundant in brain, heart, skeletal muscles and immune cells (Braverman and Moser, 2012). These vinyl-ether bonded lipids are also richly distributed in food products including fish, mollusk, livestock and poultry (Yamashita et al., 2016; Wu et al., 2019), however they have not been reported in plants or fungi yet (Braverman and Moser, 2012). Of interest, they have a multistage evolutionary history emphasizing their first appearance in anaerobic bacteria and absence in most aerobic bacteria and re-appearance in protozoa and animals (Goldfine, 2010). Plasmalogens also have been found accounting for 21–24 mol % of total phosopholipids in the slime molds (Physarum polycephalum) (Poulos et al., 1971), belonging to the class of Myxomycetes (Fiore-Donno et al., 2008).

Figure 1 summarizes a variety of proposed biological functions for plasmalogens as antioxidants in addition to their modulatory role in membrane structure and dynamics (Bozelli et al., 2021). The high susceptibility of the vinyl-ether bond at sn-1 position to radicals including reactive oxygen species (ROS) and reactive nitrogen species (RNS) as well as to the traces of acids supports their important role as a first-line defense system in biology against oxidative damages (Zhuo et al., 2021; Bozelli et al., 2021). Previously, Deng and colleagues have proposed that plasmalogens carrying PUFA chains may promote intracellular cubic membranes (CM) formation (Deng et al., 2009) with the implication in virus-induced host CM formation (Deng et al., 2010; Deng and Angelova, 2021). In addition, PUFA-plasmalogens may also act as an integrated antioxidant defense system to provide a protective shelter for nucleic acids (RNAs) and other biomolecules (Almsherqi et al., 2008; Deng and Almsherqi, 2015). It has been reported that plasmalogens are highly concentrated in lipid bilayer microdomains in cellular and subcellular organelle membranes (Messias et al., 2018). They naturally participate in multiple cellular processes including membrane fusion (Zhuo et al., 2021), cholesterol homeostasis (Honsho et al., 2015), ion transport (Messias et al., 2018) and immunomodulation (Deng and Angelova, 2021). In lipid biosynthesis, plasmalogens can be the source of supply for the precursor of PAF (Dorninger et al., 2020) (Figure 1). The modulatory role of plasmalogens in membrane dynamics mainly relies on their preference for the formation of non-lamellar inverted hexagonal (H_II_) structures (Lohner, 1996) and cubic phases (Angelova et al., 2021). Their role in membrane fusion and fission processes has been suggested and reviewed (Koivuniemi, 2017; Dean and Lodhi, 2018). Plasmalogens may also serve as a reservoir of omega-6 and/or omega-3 PUFAs whose metabolites are important in various cell signaling pathways (Messias et al., 2018; Dorninger et al., 2020). Moreover, plasmalogen has shown its anti-inflammatory effect both *in vitro* and *in vivo* (Sejimo et al., 2018)*.*

The biosynthesis of plasmalogens starts in the subcellular organelle peroxisome and completed in the endoplasmic reticulum (ER) (Rangholia et al., 2021). Plasmalogen deficiency has been reported to be associated with several human diseases as well as aging (Bozelli et al., 2021; Pham et al., 2021). Low levels of plasmalogens have been manifested in Zellweger Syndrome (ZS) (Heymans et al., 1983,^1984^), and Rhizomelic Chondrodysplasia Punctata (RCDP) (Huffnagel et al., 2013; Buchert et al., 2014), both belong to the peroxisome biogenesis disorders (PBDs) (Wanders and Waterham, 2006). Reduced levels of plasmalogens have been found in the brain and serum of patients with neurodegenerative diseases including Alzheimer’s disease (AD), Parkinson’s disease (PD), Multiple Sclerosis (MS), depression and Niemann-Pick type C disease (Schedin et al., 1997; Dragonas et al., 2009; Wood et al., 2016; Bozelli et al., 2021; Rangholia et al., 2021). Plasmalogens deficit is also implicated in other neurological disorders. For instance, the total level of plasmalogens is reduced by 15–20 % in the plasma of autistic patients (Dorninger et al., 2017). Similarly, pPE levels are decreased by 15% in the brain of autism rat model (Thomas et al., 2010). A significant drop of plasmalogens levels has been reported in the red blood cells and fibroblasts of schizophrenia patients as well (Thomas et al., 2010). Plasmalogens deficiency may be a secondary effect outcome of metabolic and inflammatory disorders including cancer, diabetes mellitus, various cardiac and respiratory diseases (Braverman and Moser, 2012; Pham et al., 2021). Plasmalogen supplementations have been reported to improve cognition (Hossain et al., 2018) and inhibit oxidative damage, neuro-inflammation and apoptosis (Che et al., 2018; Bozelli et al., 2021). Restoring plasmalogens levels has been achieved by the use of plasmalogen replacement therapy (Bozelli and Epand, 2021), which turned out to be a successful way to restore plasmalogen level as well as to improve diseased conditions *via* the potential anti-inflammatory property of plasmalogens (Bozelli et al., 2021).

## Chemical Analogs of Plasmalogen and PAF

The ether-bonded phospholipid PAF has attracted much attention (Dorninger et al., 2020), similarly to its precursor vinyl-ether bonded plasmalogen derivatives (Braverman and Moser, 2012; Pham et al., 2021), due to their importance in cell signaling, neurodegeneration, and severe coronavirus COVID-19 disease manifestations (Demopoulos et al., 2020; Deng and Angelova, 2021).

**FIGURE 2:**
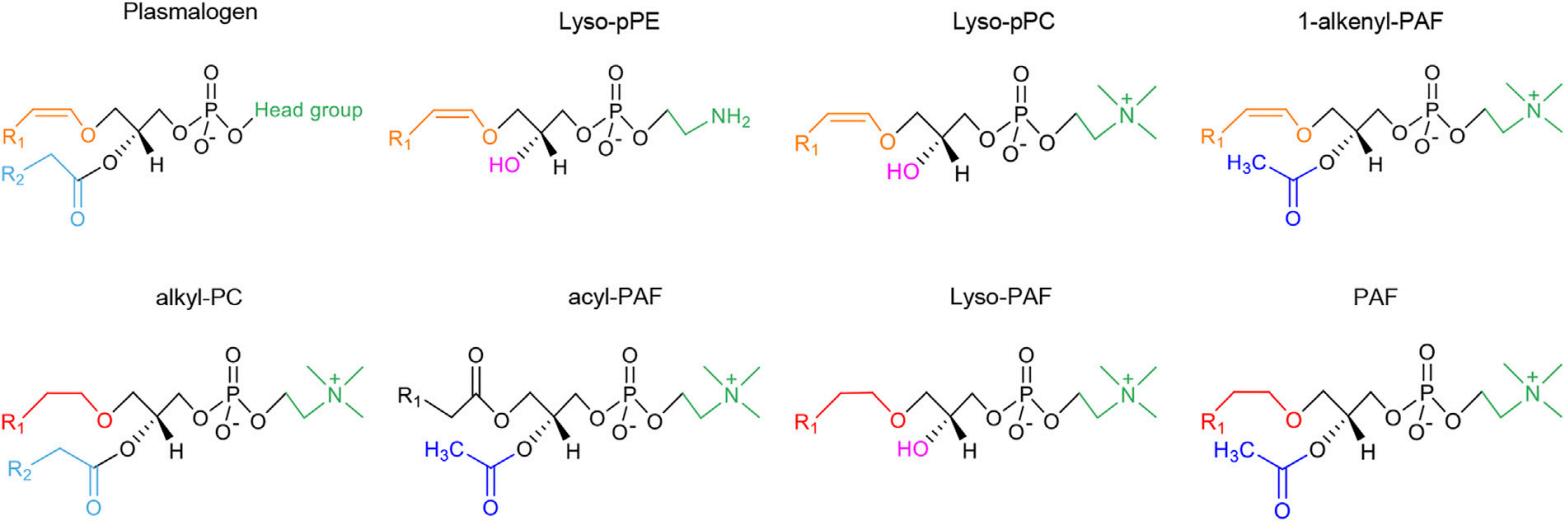
The chemical structures of vinyl ether-bonded plasmalogen lipids as compared to the family of plasmalogen analogs and plasmalogenic analogs of PAF. The head group of plasmalogen at the sn-3 position of glycerol backbone can be either ethanolamine or choline, designated as pPE or pPC, respectively. The lyso forms of plasmalogen are labeled as lyso-pPE and lyso-pPC. The chemical structures of plasmalogens (pPE and pPC) and lyso-plasmalogens (lyso-pPE and lyso-pPC), PAF and its analogs (1-alkenyl-PAF, alkyl-PC, acyl-PAF and lyso-PAF) are all structurally related. The different colors represent the specific chemical structure and functional group: alkyl groups (ether-bond) at sn-1 position are marked in red color; alkenyl groups (vinyl ether-bond) are marked in orange color; acyl groups (ester-bond) at sn-2 position are marked in blue (acyl groups are light blue while acetyl groups are dark blue); the lyso form (hydroxyl-bond) are marked with pink color; and the head groups at sn-3 position are green. R_1_ represents the alkyl chains. The saturated or unsaturated fatty acyl chains are labeled as R_2_, which is usually PUFA in plasmalogens and alkyl-PC, an acetyl group in PAF, acyl-PAF and 1-alkenyl-PAF, or a hydroxyl group in lyso-plasmalogens and lyso-PAF. The head groups of PAF and plasmalogenic analogs of PAF are often choline type, but can be ethanolamine type for pPE or lyso-pPE. In the upper row of the figure, the molecular structures are considered as plasmalogen analogs. In the lower row, the molecular structures are more similar to PAF, and therefore termed as plasmalogenic analogs of PAF.

Figure 2 summarizes the chemical structures of plasmalogens (pPE and pPC), lyso-plasmalogens (lyso-pPE and lyso-pPC), PAF and its analogs (1-alkenyl-PAF, alkyl-PC, acyl-PAF and lyso-PAF). The latter are similar to PAF, with differences as the vinyl ether bond or the ether bond at sn-1 position, and specific chemical groups at sn-2 position while PAF includes an alkyl ether bond. In the following, 1-alkenyl-PAF (PAF-like molecule with vinyl ether bond at sn-1 position) is referred to as a plasmalogenic analog of PAF. This designation was first termed by Kulikov and Muzia (1992) in their study in the effect of acyl-PAF and vinyl-PAF (1-alkenyl-PAF) on the PAF-platelet interaction. Thus, the polar lipid molecules shown in Figure 2, which are structurally similar to plasmalogen or PAF, are considered as plasmalogen analogs or plasmalogenic analogs of PAF, respectively.

**FIGURE 3:**
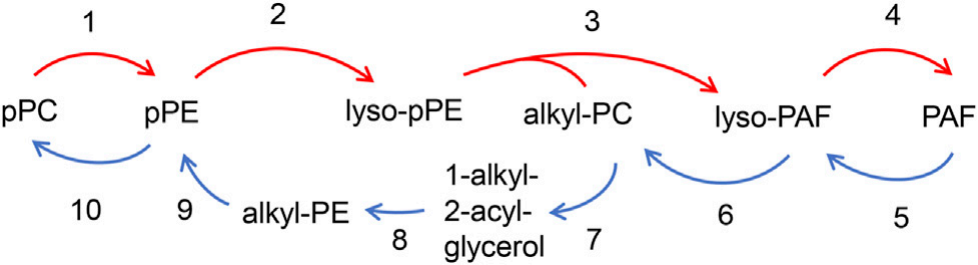
Interconversion pathway between plasmalogen and PAF. pPC can interconvert with pPE *via* head group transfer (reaction 1 and 10), Plasmalogen PE (pPE) is hydrolyzed by PLA_2_ to form lyso-pPE (reaction 2). The lyso-PE can be further reacylated by CoA-IT to form lyso-PAF (reaction 3) with the presence of alkyl-PC, and is subsequently converted to PAF by lyso-PAF AcT (reaction 4). PAF can be converted to pPE through the several steps. Losing its acetyl group by PAF-AH (reaction 5) forms lyso-PAF, which can be further converted to alkyl-PC by LPCAT (reaction 6). The alkyl-PC might be hydrolyzed by PLC to form 1-alkyl-2-acyl-glycerol (reaction 7), which then is converted to alkyl-PE by E-PT (reaction 8), and alkyl-PE is further converted to pPE with Δ1 desaturase (reaction 9).

Figure 3 depicts the interconversion pathway between plasmalogen and PAF. Plasmalogens may supply as lipid source of precursors for the generation of eicosanoids and PAF (Toyoshima et al., 1995). Evidence shows that interconversion beween pPC and pPE can be through the head group transfer (Nagan and Zoeller, 2001) (reaction 1 and 10). Lyso-pPE can be generated through the hydrolytic cleavage of pPE by phospholipase A_2_ (PLA_2_, EC 3.1.1.4, reaction 2, red arrow). The lyso-pPE can be reacylated by a coenzyme A-independent transacylase (CoA-IT, reaction 3, red arrow) (Uemura et al., 1991), and the donor in reaction 3 is alkyl-PC. The latter reaction may result in the production of lyso-PAF that can be further converted to PAF by acetyl-CoA: lyso-PAF acetyltransferase (lyso-PAF AcT, EC 2.3.1.67, reaction 4, red arrow) (Shindou et al., 2007; Goracci et al., 2009). The remodeling pathway of PAF biosynthesis is considered to be responsible for the pro-inflammatory behavior of PAF in response to acute and/or chronic inflammation (Lordan et al., 2019).

PAF can be converted to pPE *via* a series of enzymatic reactions (Frenkel and Johnston, 1992; Lee et al., 1992) (Figure 3). *In vivo* pPE can be converted into pPC through the head group transfer (reaction 10, blue arrow in Figure 3) (Nagan and Zoeller, 2001). PAF may lose its acetyl group by PAF acetylhydrolase (PAF-AH, EC 3.1.1.47, reaction 5, blue arrow) to form lyso-PAF, which can be further converted to alkyl-PC by the enzymatic action of lysophosphatidylcholine acyltransferase (LPCAT, EC 2.3.1.23, reaction 6, blue arrow) (Snyder, 1995; Shindou et al., 2007). The 1-alkyl-2-acyl-glycerol is formed by the hydrolytic cleavage of alkyl-PC at the sn-3 position by phospholipase C (PLC, EC 3.1.4.3, reaction 7, blue arrow), resulting in alkyl-PE geneartion by a ethanolamine-phosphotransferase (E-PT, EC 2.7.8.1, reaction 8, blue arrow). The alkyl-PE can be further converted to pPE with Δ1 desaturase (EC 1.14.19.77, reaction 9, blue arrow) (Maeba et al., 2018). Both direction of chemical reaction processes, namely pPC to pPE, lyso-PAF to alkyl-PC and PAF to lyso-PAF, are reversible (Figure 3). The relation between PAF and plasmalogen is most likely interconvertible and therefore several potential analogs of PAF and plasmalogen might be generated as shown in Figure 3.

## Significance of PAF as a Bioactive Alkyl-Ether Lipid

PAF is a pro-inflammatory lipid mediator with well-known messenger functions (Damiani and Ullrich, 2016). Compounds, which inactivate PAF-R, can act as PAF-R inhibitors. Demopoulos and colleagues have discovered the chemical structure of PAF and have synthesized it for confirmation using plasmalogen with a semi-synthetic method, and the alkenyl ether double bond at the sn-1 position is chemically converted into ether bond through catalytic hydrogenation (Demopoulos et al., 1979). Structurally, PAF is characterized by an alkyl ether linkage at sn-1 position, acetyl groups at sn-2 position, and a phosphocholine group at sn-3 position of the glycerol backbone (Demopoulos et al., 1979) (Figure 2). At variance, the potential plasmalogen analogs of PAF have a vinyl-ether bond at sn-1 position and a PUFA chain at sn-2 position (Figure 2). PAF analog per se has been commonly referred to as phospholipids similar in chemical or spatial structure as PAF. Notably, these PAF analogs may compete for binding to the PAF-R and are collectively known as PAF-like lipids or PAF-agonists (Lordan et al., 2019; Tsoupras et al., 2018) (see Figure 2). PAF is produced by a plethora of blood and immune cells including platelets, neutrophils, monocytes/macrophages, lymphocytes, basophiles, eosinophils and mast cells (Papakonstantinou et al., 2017). They can further act back to stimulate the cells of its origin *via* autocrine action (Demopoulos et al., 2020).

The pathophysiological role of PAF is primarily determined by its produced amount *via* lipid biosynthesis and by the extent of its enzymatic regulation. There are two enzymes that regulate the PAF activity (Figure 3), namely acetyl transferase (PAF-AT) and acetyl hydrolase (PAF-AH). The latter is a subtype of the PLA_2_ enzyme (Palur Ramakrishnan et al., 2017; Papakonstantinou et al., 2017). The homeostatic level of PAF present in plasma and biological tissues, is regulated by the balance between its anabolic and catabolic pathways (Tsoupras et al., 2018). There are two synthetic pathways of PAF, namely “*de novo*” and “*re-modeling*” pathways (Liu et al., 2017; Tsoupras et al., 2018; Rangholia et al., 2021). The latter is considered as the main pathway of PAF biosynthesis in response to inflammatory stimuli.

PAF may participate in multiple cellular processes, including inflammation, apoptosis, reproduction, angiogenesis, and glycogen degradation in addition to its physiological roles in brain function, lung maturation, regulation of blood circulation, blood pressure and coagulation (Tsoupras et al., 2018; Lordan et al., 2019). Under the diseased conditions, the excess PAF may act as a potent pro-inflammatory mediator, involved in a variety of chronic inflammatory diseases, including cardiovascular diseases, atherosclerosis, diabetes, neurodegenerative disorders and cancers. Moreover, several recent reports have suggested the implication of PAF in viral infections such as HIV (Papakonstantinou et al., 2017; Lordan et al., 2019) and even COVID-19 pathogenesis (Demopoulos et al., 2020; Klein et al., 2021).

## PAF/anti-PAF Signaling Cascades in Inflammatory Responses

Human and guinea pig PAFRs consist of a single polypeptide chain composed of 342 amino acids with seven transmembrane domains, with the characteristics of G-protein coupled receptors (GPCRs) superfamily (Chaudhary and Kim, 2021; Ishii et al., 2002). GPCRs are the largest and most diverse group of membrane receptors in the eukaryotes. These cell surface receptors act like sensors for receiving the information in the form of light energy, peptides, proteins, lipids, and sugars (Ritter and Hall, 2009), and they are the molecular targets for nearly half of the therapeutic drugs prescribed worldwide (Bridges and Lindsley, 2008). Approximately 1,000 members of the GPCRs family exhibit a conserved 7-transmembrane domain topology and can be divided into 3 main subfamilies, termed A, B and C, based on sequence similarity. The canonical view of how GPCRs may modulate cellular physiology is that the binding of ligands (such as hormones, neurotransmitters or sensory stimuli) induces the conformational changes of transmembrane and intracellular domains of the receptor, further allowing interactions with heterotrimeric G proteins (Ritter and Hall, 2009). Up to July 2021, there are total 99 GPCR structures deposited in the Protein Data Bank (PDB: www.pdbus.org), and most of them were determined by the cryo-EM method (García-Nafría and Tate, 2021). After binding to PAF-R, PAF may activate intracellular signaling pathways, including NF-κB and MAPK pathways (Lordan et al., 2019). These important inflammatory signaling pathways are initiated in macrophages (Jeong et al., 2016). They may further trigger the expression and release of a wide range of PAF-mediated inflammatory factors such as tumor necrosis factor (TNF)-α, interleukin (IL)-6, and IL-1β. All together they may orchestrate the inflammatory responses (Jeong et al., 2016). Particularly, NF-κB pathway is one of the key transcriptional pathways in PAF-mediated inflammatory response associated with the regulation of pro-inflammatory factors expression (Jeong et al., 2016). It has been demonstrated that both endogenous and exogenous anti-PAF (PAF antagonists) may inhibit the PAF activities (Lordan et al., 2019). The absence of circulating anti-PAF in the blood may result in an increase of the PAF activity and further worsen the situations of inflammation. The PAF antagonists may halt or diminish the expressions of pro-inflammatory mediators at different levels (Lordan et al., 2019).

There is a vast number of natural and synthetic anti-PAF compounds known to inhibit PAF activity and act as potential anti-inflammatory agents. The anti-PAF compounds of synthetic origin, such as statin drugs (Tsantila et al., 2011), thiazolium derivative (CV-3988), thienodiazepine derivatives such as brotizolam, WEB 2086 (apafant), and WEB 2170 (bepafant) or the natural origin, such as Ginkgolides (Papakonstantinou et al., 2017), may competitively or non-competitively inhibit PAF activity through binding to the active site of PAF-R on the cell membrane, and therefore, directly inhibit PAF signaling cascades (Papakonstantinou et al., 2017). The anti-PAF may exert anti-inflammatory effects by impeding the binding of PAF to PAF-R. This may result in the down-regulation of pro-inflammatory mediators and cytokine production *via* inhibition of NF-κB and/or MAPK pathways (Singh et al., 2013; Jeong et al., 2016; Zhaocheng et al., 2016; Li et al., 2017; Sarkar et al., 2020). It has been reported that other PAF agonists may also indirectly affect the PAF/anti-PAF signaling cascades by affecting the upstream and/or the downstream of nearby microenvironment of the PAF-R in the cell membrane or of other related membrane receptors (Tsoupras et al., 2018).

## Plasmalogenic Analogs of PAF as Potential anti-PAF and Anti-inflammation Agents

Oxidation of PC phospholipids (including pPC) may generate a series of lyso-phospholipids. Lyso-pPC carrying PUFA, usually arachidonic acid (AA), can be further fragmented to shorter-chain-length fatty acid (McIntyre et al., 1999). Some of these oxidized phospholipids carrying very short sn-2 residues (among other structural features) make them recognizable by PAF-R receptor (Zimmerman et al., 2002). There is a strong preference for PAF-R to bind PAF-like lipid molecules with ether bond at sn-1 position, acetyl residue at sn-2 position, and choline head group at sn-3 position (McIntyre et al., 1999). Plasmalogens, especially pPC (1-alkenyl PC), together with its hydrolysated form of lyso-pPC, are important polar phospholipids with similar spatial structure to PAF. The structural match between pPC/lyso-pPC and PAF may determine the high affinity to the same binding site at PAF-R (Snyder, 1990). Either pPC or lyso-pPC is potentially as a PAF analog that may compete with PAF for binding to PAF-R.

In this way, plasmalogenic analogs may effectively inhibit PAF-induced platelet aggregation through competitive binding to the receptor PAF-R (McManus et al., 1993; Smaragdi Antonopoulou and Demopoulos, 2008). It has been reported that 1-alkenyl-PAF, a plasmalogenic analog of PAF (similar to pPC or lyso-pPC), exhibited high anti-inflammatory activity in two inflammatory models of rat paw edema (Kulikov and Muzya, 2002). The authors have examined the mechanism of interaction of the plasmalogenic analogs of PAF with human platelets (Kulikov and Muzya, 1999). Of interest, 1-alkenyl-PAF has been established to act as an inhibitor of PAF-induced platelet aggregation without the influence on ADP- or thrombin-induced platelet aggregation (Kulikov and Muzya, 1999; Lordan et al., 2017). The acyl-PAF has been reported to act as potential anti-inflammatory molecules to suppress the action of PAF (Chaithra et al., 2018), Therefore, we speculate plasmalogens may be also work as a potential anti-PAF lipid compound.

Here we emphasize that plasmalogens and some plasmalogenic analogs of PAF (Figure 2) may act as novel PAF antagonists, which play an important role in modulating PAF/anti-PAF signaling cascades with implication in inflammation and inflammation-mediated disease processes (Kulikov and Muzya, 1999; Lordan et al., 2017). More about the anti-PAF effect of plasmalogen together with their analogs may inspire more future studies to approve or disapprove the hypothesis.

Plasmalogen has been demonstrated to exert an anti-inflammatory activity (Sejimo et al., 2018; Ali et al., 2019), to ameliorate neurotoxicity and inhibit neuro-inflammation and neuronal apoptosis (Yamashita et al., 2016; Che et al., 2018). A recent report showed that the intake of pPE with vinyl ether linkages at sn-1 and omega-3 PUFA at sn-2 position efficiently inhibited the downstream inflammatory and apoptotic signaling cascades in a human colon (Nguma et al., 2021). The intracellular anti-apoptotic effect of pPE has been achieved through suppressing the generation of pro-inflammatory cytokines and pro-apoptotic factors (Nguma et al., 2021).

PUFAs carried by plasmalogens at sn-2 position, especially omega-3 long-chain PUFAs, are good ligands for peroxisome proliferator-activated receptors (PPARs). These PUFAs may effectively reduce inflammatory responses through activating the PPARs proteins (Deplanque et al., 2003; Farooqui et al., 2007; Korbecki et al., 2019). Moreover, PUFAs can easily be oxidized to further activate PPARs (Echeverría et al., 2016) and form a heterodimer with the 9-cis retinoic acid receptor (RXR) (D'Angelo et al., 2018) and modulate the transcription of the target genes. In brief, plasmalogens carrying omega-3 long-chain PUFAs may provide anti-inflammatory activity through: 1) inhibiting the expression of transcription factors (*e.g.,* NF-κB), intracellular signaling proteins (*e.g*., MAP kinases) and inflammatory mediators (Carvalho et al., 2021); 2) reducing the level of reactive oxygen species (ROS) by upregulation of antioxidant enzyme expression (Carvalho et al., 2021); and 3) inhibiting microglial activation and generation of pro-inflammatory factors (Yan et al., 2003).

Plasmalogen and cholesterol are both enriched in microdomains of cell membranes. Thus, there may exist a metabolic reciprocity between them (Paul et al., 2019). Plasmalogens are proved to play important roles at multiple steps in cholesterol homeostasis *via* regulation of membrane cholesterol esterification and transportation (Mandel et al., 1998; Munn et al., 2003; Mankidy et al., 2010). The esterification of cholesterol is also dependent on the amount of pPE present in the membranes (Mankidy et al., 2010). A plasmalogen-deficient cell has lower esterified cholesterol and lower rate of HDL-mediated cholesterol efflux compared to normal wild-type cell (Mandel et al., 1998) in addition to the reduced synthesis of cholesterol (Mankidy et al., 2010). Nevertheless, these impairments are repaired by restoring the level of plasmalogens (Honsho and Fujiki, 2017), suggesting that cholesterol homeostasis is tightly regulated by plasmalogen metabolism.

Statin drugs can lower the biosynthesis of both cholesterol and farnesol (Honsho and Fujiki, 2017), the latter being involved in PAF/anti-PAF signaling cascades (Stancu and Sima, 2001). Statins have pleiotropic functions beyond cholesterol reduction, such as improvement of endothelial function, thrombogenesis reduction, and protection against oxidative stress and inflammation (Harada et al., 2015). In addition, statins may exhibit anti-PAF activity and suppress PAF biosynthesis *via* the “*de novo*” pathway *in vivo* (Tsantila et al., 2011). Hence, plasmalogens have been suggested as an alternative of statin drugs to reduce cholesterol levels (Mankidy et al., 2010; Deng and Angelova, 2021). Of interest, PUFA-pPE precursors are approximately twice as effective as statins at lowering cholesterol levels (Mankidy et al., 2010). Both outcomes of lowering cholesterol and anti-inflammation can be achieved by either plasmalogens or statins. In addition, plasmalogen is a natural product that may avoid the side effects of statin drugs, including hepatotoxicity and increased risk of diabetes mellitus (Sirtori, 2014). Thus, plasmalogens are potentially safe and efficient candidates as PAF antagonists (anti-PAF) compared to statin drugs.

## Conclusion

**FIGURE 4:**
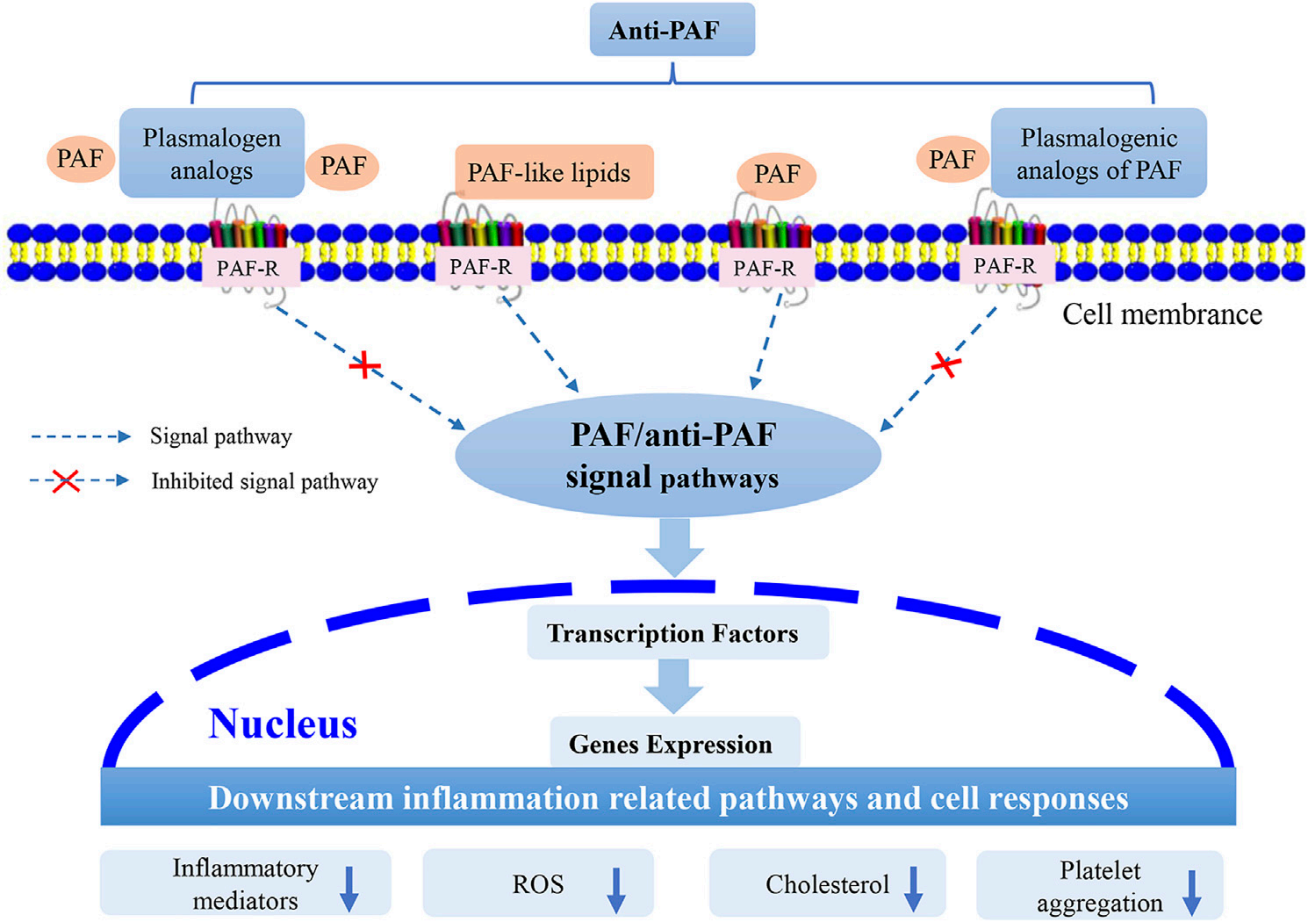
Suggested modes of action of plasmalogen, plasmalogen analogs and plasmalogenic analogs of PAF as PAF antagonists (*i.e.,* anti-PAF). Plasmalogen and its analogs, as well as plasmalogenic analogs of PAF, may compete with the other PAF lipid family (PAF and PAF-like lipids) to bind to the membrane receptor, PAF-R. Consequently, they may modulate the PAF/anti-PAF signaling pathways, such as NF-κB and MAPK, and further inhibit the expression of transcription factors and genes. The potential PAF antagonists (anti-PAF) may modulate the downstream inflammation-mediated pathways and other cellular responses.

Plasmalogens, especially pPC, lyso-pPC and other plasmalogenic analogs of PAF (Figure 2), are characterized by the similar chemical structures as PAF, and may be considered as natural PAF analogs acting as anti-PAF compounds (PAF antagonists). This structural analogy is suggested to be beneficial for modulation of inflammatory/anti-inflammatory responses. Plasmalogens and plasmalogenic analogs of PAF may compete with PAF or PAF-like molecules (PAF agonists) for binding to the PAF-R (Figure 4). As a consequence, they may modulate PAF/anti-PAF signaling cascades and further re-balance PAF levels in various inflammation-mediated disease processes. Plasmalogens, with a vinyl-ether bond at sn-1 position and PUFA chain at sn-2 position, reveal per se their remarkable roles as antioxidants and anti-inflammatory agents as well as regulators of cholesterol homeostasis. Plasmalogen, plasmalogen analogs and plasmalogenic analogs of PAF may thus offer novel therapeutic developments as potential anti-PAF compounds for the prevention and treatment of a variety of inflammation-mediated diseases including diabetes, cancers, cardiovascular and neurodegenerative disorders in addition to the expansive manifestations of the COVID-19 pathology (Demopoulos et al., 2020). In addition, the prevention and inhibition of neuro-inflammation is of particular interest for slowing down neuro-degeneration.

In conclusion, we propose that plasmalogens supplementation together with anti-PAF enriched food, such as the Mediterranean diet (Tsoupras et al., 2018; Detopoulou et al., 2021), may provide an alternative strategy (Bozelli and Epand, 2021) in inflammatory disease prevention and treatment through rebalancing of the inflammatory mediators and radicals, consequently return or regain system homeostasis.
